# Characteristics of bacterial community in eyelashes of patients with *Demodex* blepharitis

**DOI:** 10.1186/s13071-024-06122-x

**Published:** 2024-02-14

**Authors:** Dulei Zou, Xiuhai Lu, Fangying Song, Xiaowei Zhong, Huabo Chen, Ju Zhang, Yabin Tian, Li Pei, Fengjie Li, Xi Lu, Weiyun Shi, Ting Wang

**Affiliations:** 1 Qingdao University, Qingdao, China; 2 Eye Hospital of Shandong First Medical University (Shandong Eye Hospital), Jinan, China; 3 State Key Laboratory Cultivation Base, Shandong Provincial Key Laboratory of Ophthalmology, Eye Institute of Shandong First Medical University, Qingdao, China; 4 School of Ophthalmology, Shandong First Medical University, Jinan, China; 5 Qingdao Eye Hospital of Shandong First Medical University, Qingdao, China

**Keywords:** *Demodex* blepharitis, Eyelash, Bacterial community, *Burkholderia*

## Abstract

**Background:**

*Demodex* blepharitis (DB) is a common disease of the ocular surface. The characteristics of the bacterial community in eyelash roots after *Demodex* infestation are still unknown. Knowledge of the characteristics of the bacterial community of eyelash follicles in patients with DB can provide valuable insights for guiding the diagnosis and treatment of DB.

**Methods:**

Twenty-five patients with DB (DB group) and 21 non-DB volunteers (control group) were enrolled in the study. Eyelashes from the upper eyelid of the right eye were sampled, and 16S ribosomal DNA (rDNA) sequencing was performed to determine the V3-V4 regions of the microbial 16S rDNA gene within 1 month of infestation. The sequencing data of the two groups were analyzed and compared. The effect of the bacterium *Burkholderia* on the survival of *Demodex* mites was evaluated using *Demodex* obtained from 12 patients with DB other that the patients in the DB group.

**Results:**

A total of 31 phyla and 862 genera were identified in the DB and control groups. The five most abundant phyla in the two groups were Proteobacteria, Firmicutes, Actinobacteria, Bacteroidetes and Cyanobacteria. The abundance of Actinomycetes was significantly higher in the DB group than in the control group. At the genus level, the five most abundant genera in the two groups were *Pseudomonas*, *Burkholderia-Caballeronia-Paraburkholderia*, *Rolstonia* and *Acinetobacter*; *Clostridium sensu stricto 1* was abundant in the control group and *Corynebacterium_1* was abundant in the DB group. Compared with the control group, the abundance of *Burkholderia-Caballeronia-Paraburkholderia* was 2.36-fold lower in the DB group. Linear discriminant analysis Effect Size (LEfSe) analysis revealed *Burkholderia-Caballeronia-Paraburkholderia*, *SC_I_84_unclassified*, *Nonmyxobacteria* and *Succinvibrio* to be the major biomarkers in the control group and *Catenibacterium* and *Lachnospiraceae NK4A136 group* to be the major biomarkers in the DB group. To explore the performance of these optimal marker models, receiver operational characteristic curve analysis was performed, and the average area under the curve value of *Burkholderia-Caballeronia-Paraburkholderia* was 0.7448. *Burkholderia cepacia* isolated from normal human eyelashes was fermented, and the* Demodex* mites isolated from patient eyelashes were cultured together with its fermented supernatant. The results showed that the fermentation supernatant could significantly reduce the survival time of the *Demodex* mites, suggesting the potential therapeutic value of this bacterium against *Demodex*.

**Conclusions:**

The composition of the bacterial community in the eyelashes of DB patients differed from that in eyelashes of healthy volunteers, revealing a decrease in bacterial diversity in infested eyelashes. This decrease may be related to the occurrence and development of DB. The supernatant of *Burkholderia cepacia* culture medium was found to inhibit the growth of *Demodex* in eyelash hair follicles, providing a new insight with potential applications for the clinical treatment of *Demodex* infestation.

**Graphical Abstract:**

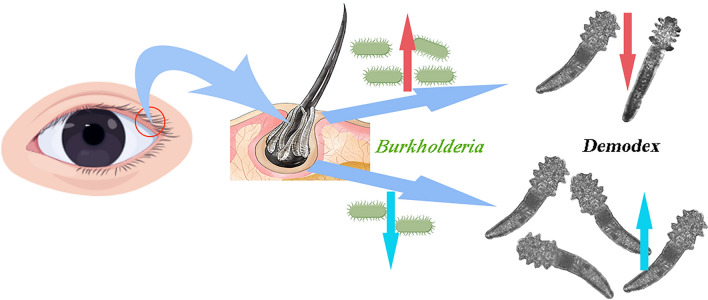

**Supplementary Information:**

The online version contains supplementary material available at 10.1186/s13071-024-06122-x.

## Background

Blepharitis is one of the most frequently encountered ophthalmic diseases in clinical practice. One study reported that 37% of patients who visited an eye doctor in the USA suffered from blepharitis [1] and that 36% of patients experiencing ocular discomfort or irritation received a diagnosis of blepharitis [2]. In cases of blepharitis, the infestation rate of mites, primarily of tiny parasitic mites belonging to the genus *Demodex*, can reach as high as 90.0% [3, 4]. Common symptoms of DB include itching, burning, dryness, tearing and blurred vision [1]. Due to the non-specific nature of its clinical manifestations, this disease is often overlooked by ophthalmologists. In addition, because the pathological processes of blepharitis induced by *Demodex* are poorly understood, therapies are limited to local cleaning and acaricidal treatments, which often yield unsatisfactory results. In some cases, patients with DB may even develop severe and refractory damage to the cornea and ocular surface [5].

The pathogenesis of DB may involve various mechanisms, such as the direct destruction and damage caused by *Demodex* [6], bacterial infection carried on the surface of *Demodex* [7–9] and immune responses triggered by bacteria inhabiting the body of *Demodex* mites [10, 11]. While antibacterial treatment has been proposed as a routine therapy for DB [1], the clinical evidence for this treatment seems to be insufficient [12]. Therefore, elucidating the characteristics of the local bacterial community in patients with DB would be beneficial for enhancing current understanding of the pathogenesis and guiding clinical treatment.

**Fig. 1:**
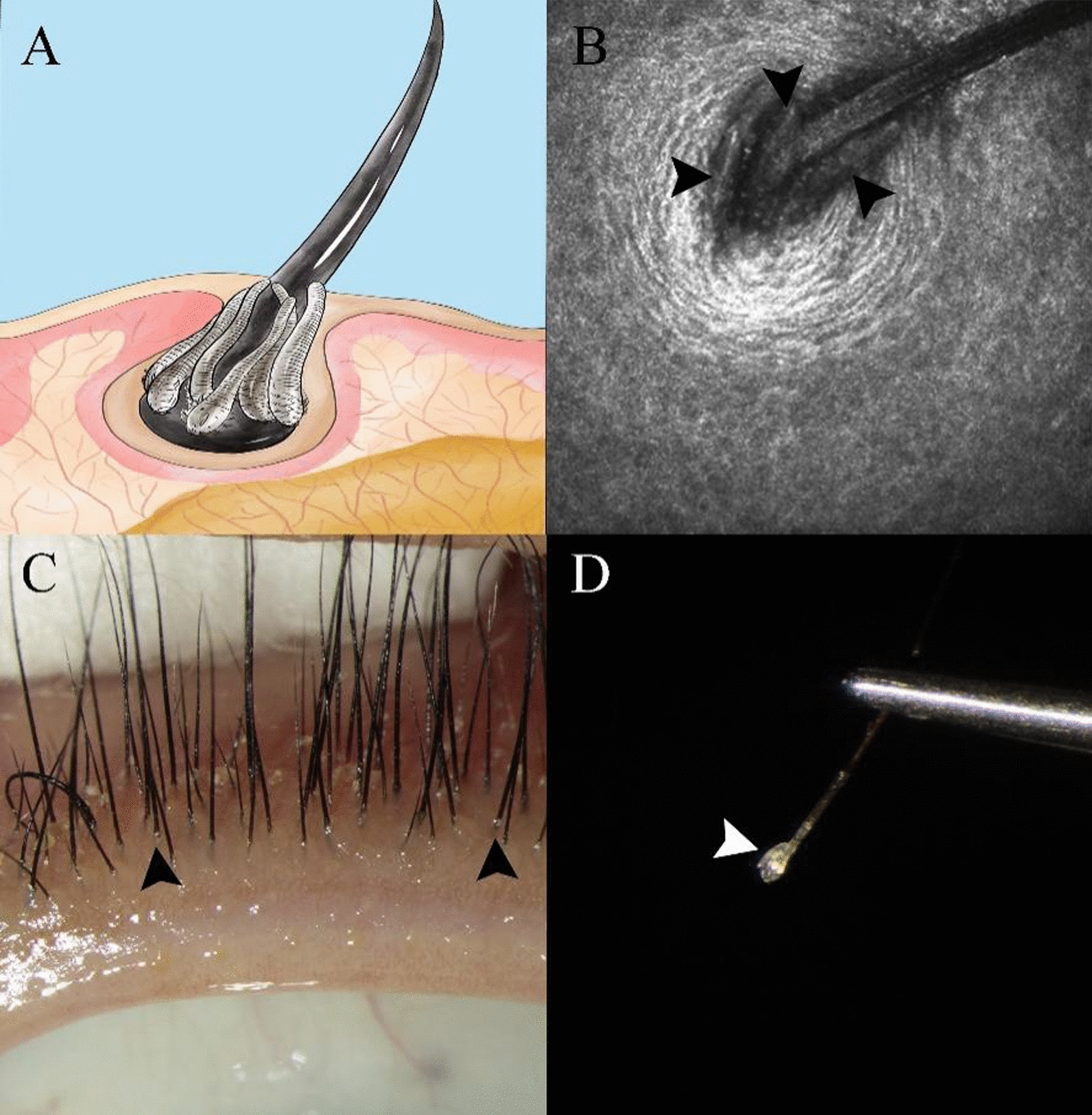
*Demodex* mites on the eyelashes of one patient with *Demodex* blepharitis (DB). **a** Schematic diagram of *Demodex* on an eyelash. **b** In vivo confocal microscopy shows that *Demodex* mites inhabit the eyelash follicle attached to the root of the eyelash (black arrowhead). **c** “Cuff-like” changes at the root of eyelashes (black arrowhead). Magnification: ×16. **d** Under a microscope, *Demodex* residing at the root of the eyelash is pulled out with a pair of tweezers (white arrow). Magnification: ×40)

*Demodex* mites mainly inhabit eyelash follicles, meibomian glands and sebaceous glands. Detecting *Demodex* in or around eyelash follicles can aid in diagnosing DB (Fig. 1a–d), but only a few studies have been reported on the bacterial community of blepharitis in which samples from eyelashes were compared to those from meibomian glands and tears [13–16], possibly due to the low level of bacteria in eyelash tissue and the limitations of traditional bacterial culture. Therefore, in the present study, our aim was to explore the characteristics of the bacterial community in samples obtained from the root of eyelashes via 16S ribosomal DNA (rDNA) sequencing technology and to determine the relationship between *Demodex* and the bacterial community of eyelash follicles.

## Methods

### Study population

A total of 25 patients with DB (DB group) and 21 healthy volunteers (control group) who were matched for age and sex were randomly enrolled for 16S rDNA sequencing of eyelash samples. The diagnostic criteria of DB were: (i) symptoms, including conjunctival hyperemia, itching, foreign body sensation and recrudescent and refractory chalazions, presenting a chronic or subacute course; (ii) cuff secretions at the root of eyelashes; and (iii) positive *Demodex* detection results when the count of *Demodex* was ≥ 3 per 3 three eyelashes from any one of the four eyelids by slit-lamp microscopy. Patients aged < 18 years or had previous eye surgery or trauma, serious diseases or eye medication in the month directly preceding enrolment were excluded from the study [1, 17].

This study complied with the tenets of the Helsinki Declaration, was reviewed and approved by the Ethics Committee of The Eye Hospital of Shandong First Medical University (Shandong Eye Hospital) and was registered on the Chinese Clinical Trial Registry (ChiCTR No: 2100041622). Written informed consent was obtained from all participants.

### Preparation of eyelash specimens

To ensure consistency, all eyelash specimens were taken from the upper eyelid of the right eye. To obtain the specimens, we first dripped one drop of 0.5% proparacaine hydrochloride (Alcon Laboratories, Fort Worth, TX, USA) into the conjunctival capsule as topical anesthesia. Type III entoiodine disinfectant (Likang, Shanghai, China) was subsequently used to clean the upper and lower eyelid margins and eyelashes, following which a pair of sterilized ophthalmic eyelash tweezers was used to randomly pull out three eyelashes from the patients with DB and the healthy volunteers. Sterile cotton swabs were used to wipe the disinfected eyelashes as blank control. The samples were quickly placed into sterile centrifuge tube without enzymes and stored at – 80 °C after being treated with liquid nitrogen. All samples were collected by the same doctor and subjected to 16S rDNA sequencing within 1 month of collection [18]. Strict attention was given to maintaining aseptic conditions during sampling and the temperature during transportation.

### Extraction and detection of bacterial DNA

Total DNA was extracted using the CTAB method. The quality of DNA was assessed by agarose gel electrophoresis, and the quantity was measured by spectrophotometry on an ultraviolet spectrophotometer. The corresponding primers were designed according to the conserved region of DNA in the microbial ribosomes to be detected, and the universal connector and barcode sequences were added. The variable regions (V3+V4) were selected to complete PCR amplification. The universal primers for the bacterial 16S rDNA (V3+V4) regions were 341F (5ʹ-CCTACGGGNGGCWGCAG-3ʹ) and 805R (5ʹ-GACTACHVGGGTATCTAATCC-3ʹ). PCR amplification was performed in a 25 μl of reaction mixture (2.5 μl of each primer, 12.5 μl of PCR Premix, 25 ng of template DNA and ddH_2_O [for volume adjustment]). The PCR cycling conditions were: an initial denaturation at 98 °C for 30 s; followed by 32 cycles of denaturation at 98 °C for 10 s, annealing at 54 °C for 30 s and extension at 72 °C for 45 s; with a final extension for 10 min at 72 ℃.

The PCR products were visualized by electrophoresis in a 2% agarose gel. Throughout the DNA extraction process, ddH_2_O was used as a negative control to control for false-positive PCR results. The PCR products purified with AMPure XT beads (Beckman Coulter Genomics, Chaska, MN, USA) were quantified with Qubit quantification assay kits (Invitrogen, Thermo Fisher Scientific, Waltham, MA, USA). The library with a concentration > 2 nM was selected for gradient dilution. The size and the quantity of the amplicon library were assessed using the Agilent 2100 Bioanalyzer (Agilent Technologies, Santa Clara, CA, USA) and the Library Quantification Kit (Kapa Biosciences, Woburn, MA, USA) for preparation for sequencing on Illumina platforms (Illumina Inc., San Diego, CA, USA), respectively. Finally, the libraries were sequenced on a NovaSeq PE250 platform (Illumina Inc.), which utilizes high-throughput sequencing technology to generate large amounts of sequence data quickly and accurately.

Samples were sequenced by LC Sciences (Houston, TX, USA) on an Illumina NovaSeq platform according to the manufacturer's recommendations. Paired-end reads were assigned to samples based on their unique barcodes and truncated by removing the barcode and primer sequence. These paired-end reads were then merged using FLASH software. Quality filtering was performed on the raw reads using specific filtering conditions to obtain high-quality clean tags, as determined by the fqtrim (v0.94) software utility. Chimeric sequences were removed using the Vsearch software tool (v2.3.4). After dereplication using the DADA2 software package, a feature table and feature sequences were obtained. Alpha diversity and beta diversity analyses were performed by randomly normalizing the sequences to the same depth. The feature abundance was then normalized using the relative abundance of each sample, as classified by the SILVA database (release 138). Alpha diversity was assessed using five indices: Chao1, Observed OTU richness, Good's Coverage, Shannon and Simpson. These indices were calculated using the QIIME2 platform. Beta diversity analysis was conducted using QIIME2, and the resulting graphs were generated using the R package. The pROC package of R language (R-3.4.4)(R Foundation for Statistical Computing, Vienna, Austria) was used for receiver operating characteristic (ROC) curve analysis. The area under the curve (AUC) criteria for drawing curves were as follows: > 0.5 indicated no consistency with the actual situation; 0.5 indicated no diagnostic value at all; between 0.5 and 0.7 indicated a low diagnostic value; between 0.7 and 0.9 indicated a certain diagnostic value; and > 0.9 indicated a high diagnostic value. Sequence alignment was performed using BLAST, and the feature sequences were annotated using the SILVA database. Other diagrams were created using the R package (v3.5.2).

### Isolation and identification of *Burkholderia* from eyelashes

Three eyelashes were taken from each volunteer and patient and placed in a sterile tube containing 500 ul of LB broth. Gram staining was used to determine the presence of colonies of bacterial growth, following which the colonies were transferred to MacConkey’s medium; species identification was based on 16S sequencing after 2 days of culture. After a 2-min electric shock, 100 μl of liquid was inoculated into each of two blood plates, followed by culture at 37 °C in a temperature chamber with 5% CO_2_ for 3 days, then in an anaerobic bag for 5 days. After the colonies developed, they were identified by Gram staining and transferred to a culture plate containing McConkey’s medium to culture. Then after culture, a small amount of the bacterial colony (200 μl) was picked with a sterile pipette tip and spread in a clockwise direction on a ground steel target (MTP384 polished steel target, Zybio Inc, Chong Qing, China). After drying, it was covered with 1 μl of formic acid, dried again and again covered with 1 μl of matrix solution. Mass spectrometry detection was performed after drying (Zybio Inc., Chongqing, China). 16S rRNA gene sequencing was performed and aligned through the National Center for Biotechnology Information (NCBI).

### Investigating the effect of *Burkholderia* on *Demodex* survival

To detect whether *Burkholderia* bacteria could influence the survival of *Demodex* mites, we cultured the *Burkholderia* isolated from eyelashes on a blood agar plate and then transferred them to a liquid medium containing 10 g/l peptone, 1 g/l yeast extract and 0.2 g/l calcium chloride after two passages. After culture in a shaking incubator at 37 °C at 200 RPM for 48 h, the fermentation supernatant of the liquid medium containing *Burkholderia* (DB group) and not containing *Burkholderia* (control group) was obtained following separation by 8000 RPM for 10 min. The *Demodex* of eyelash samples from 12 patients with DB other than those in the DB group were cultured in a 24-well plate containing 500 μl supernatant with 10 μg/ml gentamycin, with one eyelash per well, at 37 °C and examined under a microscope every 3 h. The death of *Demodex* mites was determined when the limbs remained motionless for 2 min or when the body surface was observed to shrink, become discolored and deformed and the contents were purged [19]. In order to avoid misclassification, 3 h after we initially determined that a *Demodex* mite was dead, we would observe the *Demodex* mite once again for activity, in order to confirm or modify the time of death.

### Statistical analysis

The SPSS 20.0 software package was used for statistical analyses (SPSS IBM, Armonk, NY, USA). Student’s t-test was used to compare the differences in age, sex, main indicators and *Demodex* survival time between groups. The ANOSIM (ANalysis Of Similarities) and Mann–Whitney U-tests were used to compare the alpha and beta diversity of the two groups. A *P*-value < 0.05 was considered to indicate statistical significance.

## Results

Eyelash samples for 16S rDNA sampling were collected from 25 patients with DB (10 males, 15 females) whose mean (± standard deviation [SD]) age was 35.80 ± 12.42 years, and from 21 healthy volunteers (7 males, 14 females), whose mean age was 32.71 ± 9.63 years. There was no significant difference in sex (Chi-square test, χ^2^=0.006, *df *=1, *P* = 0.938) or age (Student’s t-test, *t* = − 0.928, *P* = 0.091) between the two groups. A total of 3,867,786 raw data points were obtained from the 46 participants, of which 3,469,354 were valid (89.72%). No bacterial DNA was detected in the blank control samples.

### Bacterial community structure and abundance

**Fig. 2:**
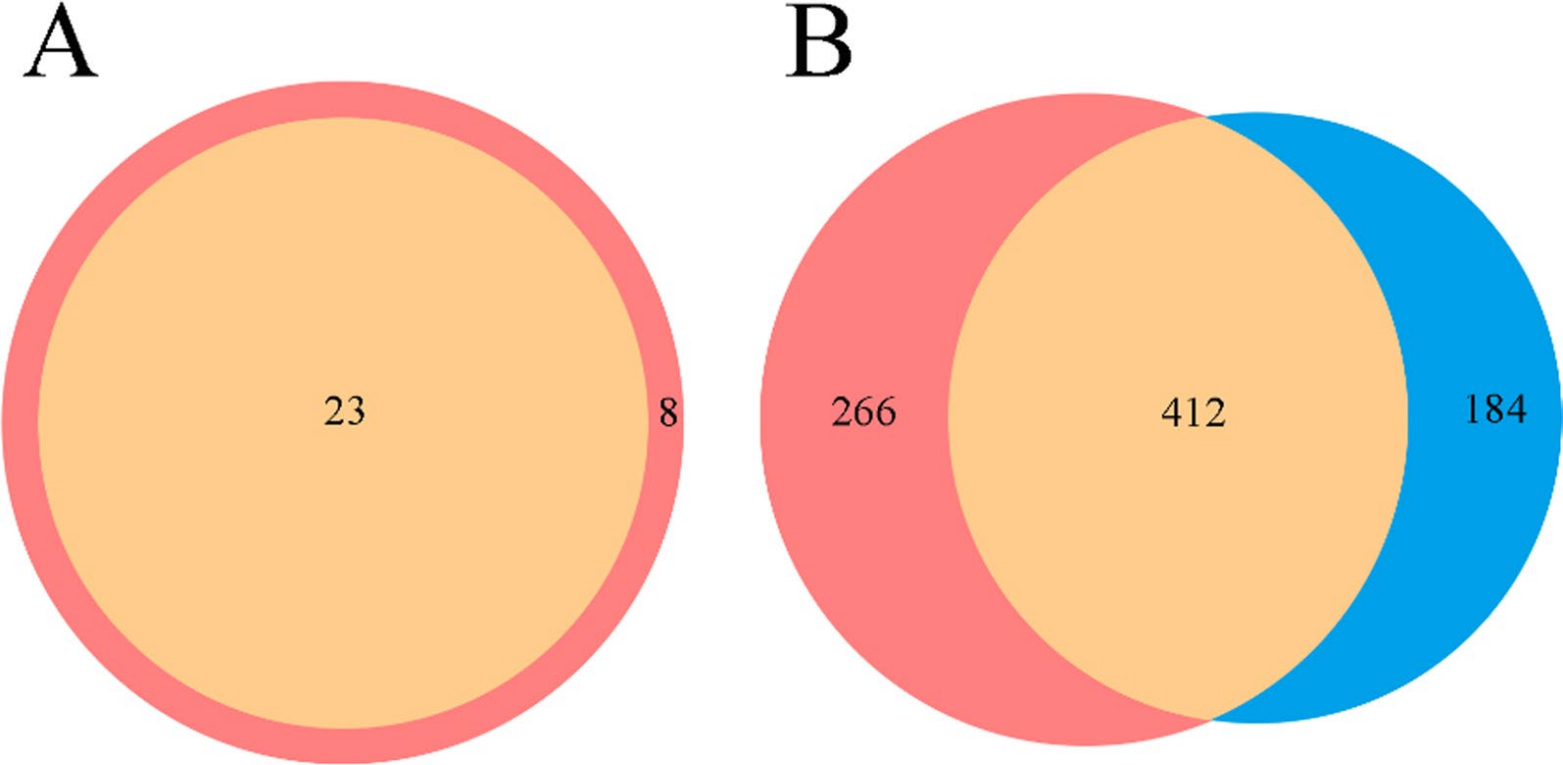
Venn diagrams of common and distinct taxa between the two study groups (DB group and healthy control group) at the phylum level (**a**) and genus level (**b**). The overlapping part of** b** represents the shared components of the DB group and the healthy control group; the non-overlapping part represents the unique components in each group. The red part of the diagram represents the control group (eyelashes from healthy volunteers); the blue part of the diagram represents the DB group (eyelashes from patients with *Demodex* blepharitis); and the brown part of the diagram represents overlap between the two groups (i.e. genera common to both study groups)

Significantly fewer bacterial phyla and classes were identified in the DB group than in the control group (Student’s *t*-test, *t* = 2.211, *P* = 0.033; Student’s *t*-test, *t* = 2.092, *P* = 0.047) (Table 1). Eight unique phyla of bacteria were identified from the eyelashes of the control group, and 23 unique phyla were identified in the DB group. At the genus level, the two groups shared 412 identical genera, while there were 266 genera unique to the control group and 184 genera unique to the DB group. Overall, there was a high degree of overlap of genera between the two groups (Fig. 2a, b).

**Table 1:** The quantity of detected bacteria in the two study groups at different taxonomic levels

Level	Control group	*Demodex* blepharitis group	*P*-value (t-test)
Phylum	12.76 ± 3.015	10.96 ± 2.406	0.033*
Class	23.76 ± 10.449	18.68 ± 4.181	0.047*
Order	48.67 ± 16.818	40.68 ± 8.868	0.060
Family	73.24 ± 24.580	63.32 ± 15.842	0.121
Genus	112.14 ± 39.311	99.08 ± 26.625	0.204
Species	138.14 ± 51.442	122.80 ± 34.521	0.252

*Significant between-group difference at *P* < 0.05

**Fig. 3:**
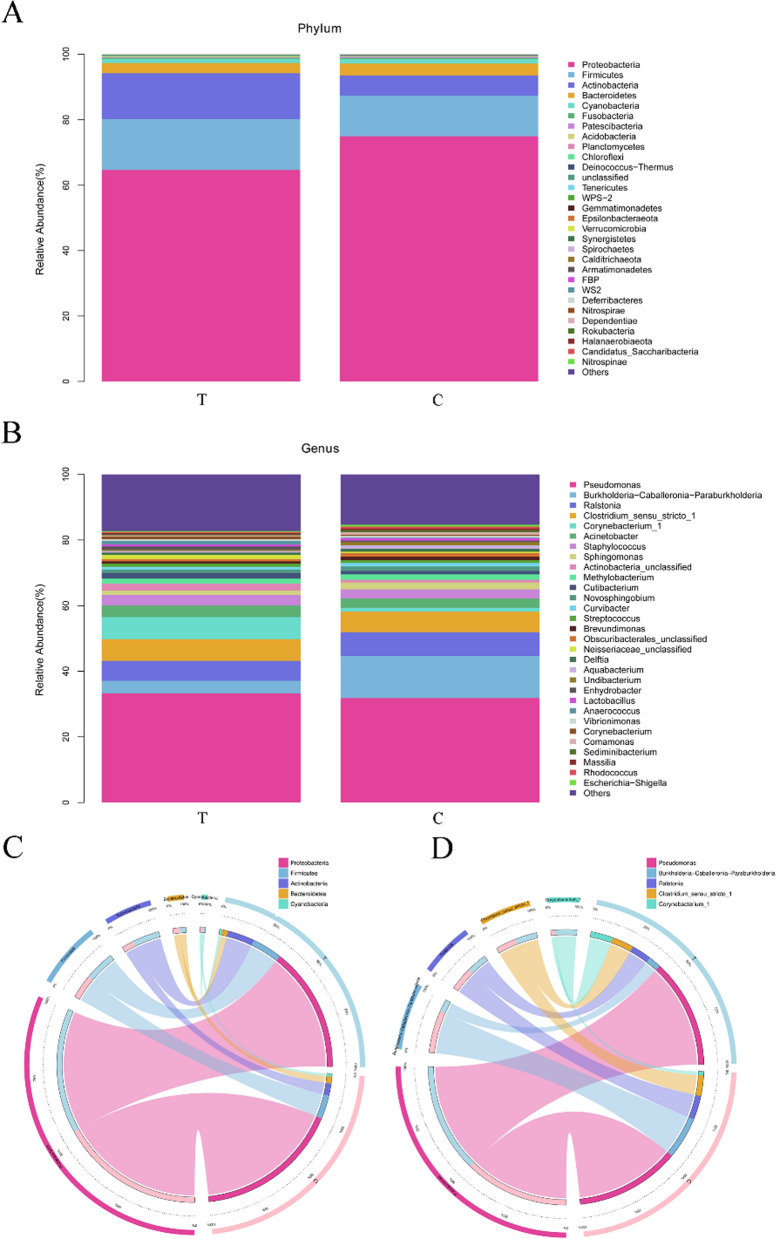
The relative abundance of bacterial community species. **a**, **b** Relative abundance of the 30 most common bacterial species in the two study groups at the *phylum* level (**a**) and *genus* level (**b**). **c**, **d** Circos circle diagram of the relative abundance of the five most common bacterial species in both study groups at the *phylum* level (**c**) and genus level (**d**). C, Control group (eyelashes from healthy volunteers); T, DB group (eyelashes from patients with *Demodex* blepharitis)

We further explored the abundance of bacterial community in the two groups and identified the top 30 most abundant bacteria at different taxonomic levels (Fig. 3). The five most common bacterial phyla in both the control and DB groups were Proteobacteria (74.96% vs. 64.66%, respectively; Student’s *t*-test, *t* = 1.326, *P* = 0.192), Firmicutes (12.38% vs. 15.58%, respectively; Student’s *t*-test, *t* = − 0.560, *P* = 0.579), Actinobacteria (6.21% vs. 13.98%, respectively; Student’s *t*-test, *t* = − 2.213, *P* = 0.035), Bacteroidetes (3.62% vs. 3.13%, respectively; Student’s *t*-test, *t* = 0.528, *P* = 0.600) and Cyanobacteria (1.40% vs. 1.27%, respectively; Student’s *t*-test, *t* = 0.235, *P* = 0.815). The abundance of Proteobacteria, Bacteroidetes and Cyanobacteria was lower in the DB group than in the control group, but the difference was not significant (*P* > 0.05, Student’s *t*-test). In addition, the abundance of Firmicutes and Actinomycetes increased in the DB group, with the abundance of Actinomycetes increasing significantly (*P* < 0.05, Student’s t-test).

In the control group, the five most common bacterial genera in the control group were *Pseudomonas* (31.82%), *Burkholderia-Caballeronia-Paraburkholderia* (12.85%), *Rolstonia* (7.26%), *Clostridium_sensu_stricto_1* (6.26%) and *Acinetobacter* (2.83%), and in the DB group, they were *Pseudomonas* (33.29%), *Corynebacterium_1* (6.71%), *Clostridium_sensu_stricto_1* (6.62%), *Rolstonia* (6.07%) and *Burkholderia*-*Caballeronia-Paraburkholderia* (3.82%). The abundances of *Pseudomonas*, *Corynebacterium_1*, *Acinetobacter* and *Clostridium_sensu_stricto_1* were higher in the DB group than in the control group, but the difference was not significant (Student’s *t*-test, *t* = − 0.156, − 1.770, − 0.570, − 0.069, respectively; *P* = 0.877, *P* = 0.084, *P* = 0.572 and *P* = 0.945, respectively). In the DB group, the abundance of *Burkholderia-Caballeronia-Paraburkholderia* decreased by 2.36-fold (Student’s *t*-test, *t* = 3.366, *P* = 0.002) and that of *Rolstonia* was also lower (Student’s *t*-test *t* = 0.643, *P* = 0.523).

### Alpha and beta diversity

**Fig. 4:**
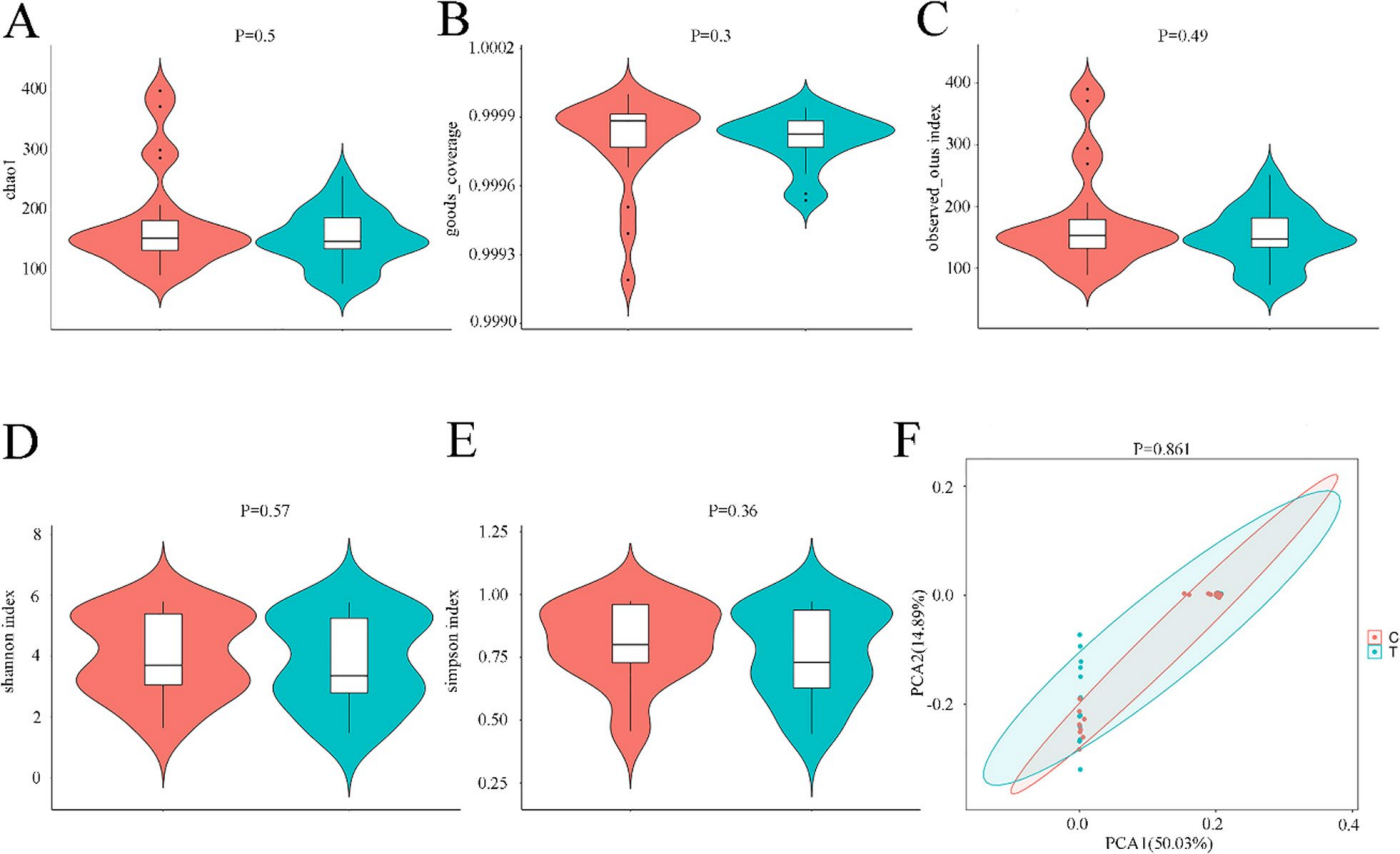
Alpha diversity and beta diversity. **a**–**e** Violin plot of alpha diversity based on diversity indexes: **a** Chao1 index, **b** Good’s Coverage index, **C** Observed OTU index, **d** Shannon index, **e** Simpson index (*P* > 0.05, Mann–Whitney U-test).** f** Beta diversity PCA. The distance between samples from the two groups was close, and no obvious separation trend was observed (*P* > 0.05, ANOSIM test). The red part of the diagram represents the control group (eyelashes from healthy volunteers); the blue part of the diagram represents the DB group (eyelashes from patients with *Demodex* blepharitis). C, Control group; PCA, principal component analysis; T, DB group

There were no significant differences in the Chao1 index, Good’s Coverage index, Observed OTU richness index, Shannon index or Simpson index between the DB group and the control group (*P* > 0.05, Mann–Whitney U-test), indicating no significant differences in sequencing depth, abundance or evenness of the bacterial community between the two groups (Fig. 4a-e). Principal component analysis revealed no significant difference in beta diversity between groups (*P* > 0.05, ANOSIM test). The separation trend of the composition of both groups was not obvious, and the composition similarity was high (Fig. 4f).

### Linear discriminant analysis Effect Size multistage species difference discriminant analysis

**Fig. 5:**
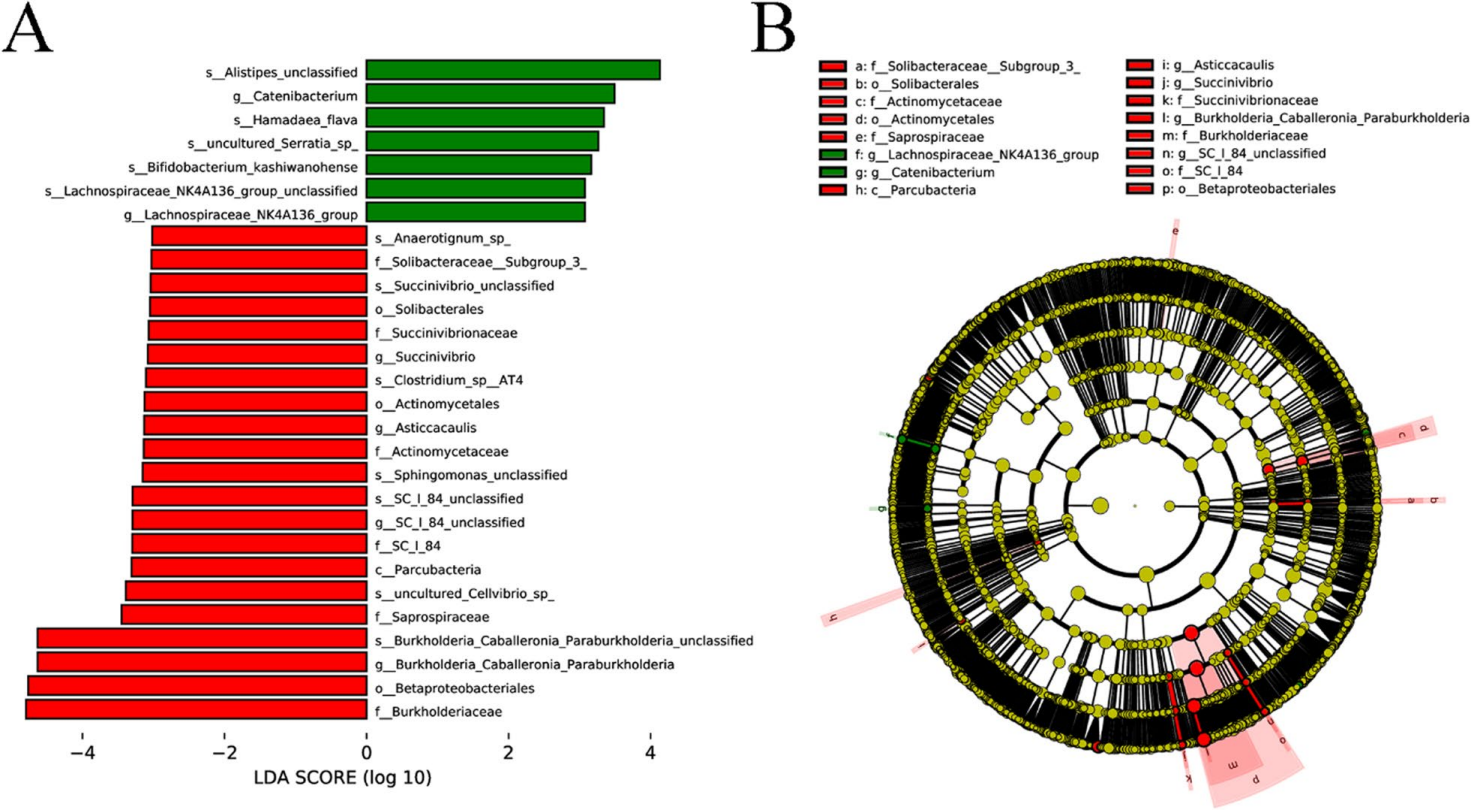
LDA Effect Size multilevel discriminant analysis of species differences. **a** Histogram of LDA score distribution shows those biomarkers that were significantly different. The LDA value represents the influence of bacterial species. **b** Cladogram. The circles radiating from the inside outwards represent taxonomic levels, from kingdom (single circle; innermost) to genus (or species). Each small circle at different classification levels represents a bacterial classification at that level, and diameter of the circle diameter is proportional to the relative abundance. Color coding: The species that are not significantly different are uniformly colored yellow; the species that are significantly different are colored with the group. The red and green nodes represent those bacterial species which play an important role in the red and green groups, respectively: C (red), Control group; T (green), *Demodex* blepharitis (DB) group. LDA, Linear discriminant analysis

The linear discriminant analysis Effect Size (LEfSe) analysis revealed that the major biomarkers at the genus level were *Burkholderia-Caballeronia-Paraburkholderia*, *SC_I_84_unclassified*, *Nonmyxobacteria* and *Succinvibrio* in the control group and *Catenibacterium* and *Lachnospiraceae_NK4A136_group* in the DB group (Fig. 5a, b). *Burkholderia-Caballeronia-Paraburkholderia* was the only dominant bacterium among the 30 most common biomarkers in the two groups.

### Genus differences between groups

**Fig. 6:**
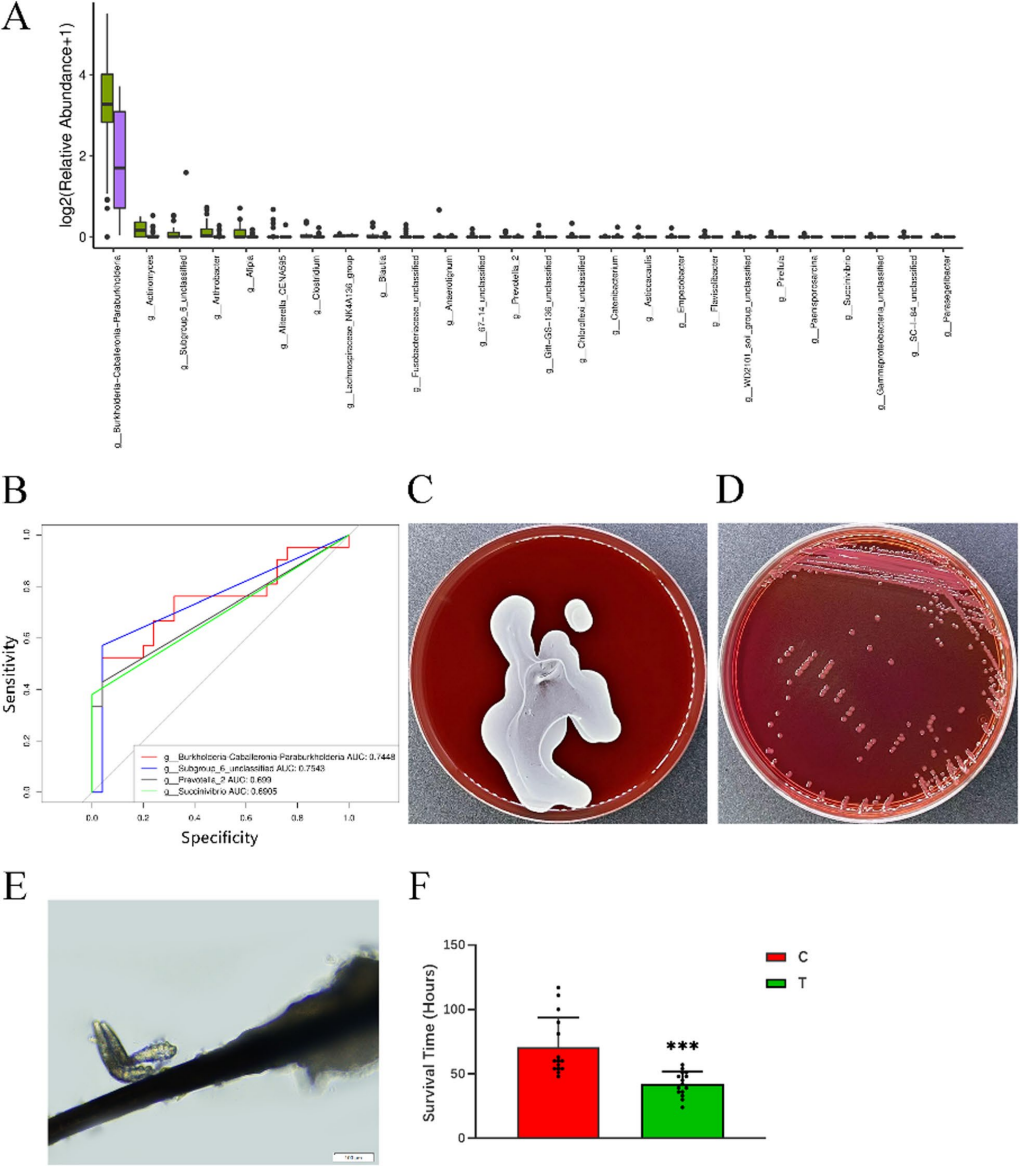
**a** Analysis of significant differences at the genus level (Mann–Whitney U-test): C (purple), Control group; T (green), *Demodex* blepharitis (DB) group. **b** ROC survival curve analysis. The four most common genera that differed between the study groups at the genus level. **c** Bacterial culture of an eyelash. **d** Morphology of *Burkholderia cepacia* colony. **e** Morphology of* Demodex* mites under the optical microscope. (bar = 100 μm). **f** Effect of the fermentation supernatant of *Burkholderia cepacia* on the survival time of *Demodex* in vitro. The fermentation supernatant of *Burkholderia cepacia* significantly shortened the survival time of *Demodex* (***P < 0.001, Student’s t-test): C (red), fermentation supernatant of medium without *B. cepacia*; T (green), fermentation supernatant of medium with *B. cepacia*. ROC, Receiver operation characteristic

We compared differences in genus composition between the two study groups based on the relative abundance table of sample species. There were 26 genera with significant differences at the genus level (*P* < 0.05, Mann–Whitney U-test, Fig. 6a). Only one of the 30 most abundant genera, *Burkholderia-Caballeronia-Paraburkholderia*, was significantly difference between the two study groups (*P* < 0.05, Mann–Whitney U-test), with a lower abundance in the DB group. The corresponding β-Proteobacteria order, *Burkholderia* family, and related species also showed differences in abundance (results not shown).

### ROC analysis

AN ROC analysis to examine the performance of these optimal marker models between two groups. The average AUC value of *Burkholderia-Caballeronia-Paraburkholderia* was 0.7448, indicating a powerful diagnostic potential for DB (Fig. 6b). The corresponding β-Proteobacteria order, *Burkholderia* family and related species also showed certain diagnostic efficacy.

### The effect of *Burkholderia* on *Demodex* survival

Eyelashes of 10 patients with DB and 10 healthy volunteers were collected for bacterial culture. The main bacterial strains obtained after culture were *Corynebacterium jeikeium*, *Corynebacterium macginleyi*, *Staphylococcus epidermidis*, *Propionibacterium acnes*, *Staphylococcus aureus*, among others (Fig. 6c). Among these samples, *Burkholderia* was successfully isolated from one of the healthy volunteers (Fig. 6d; Additional file 1). After 16S rDNA sequencing, identification and comparison, we obtained the sequence of the isolated strain (Additional file 1) and identified it as *Burkholderia cepacia *(Accession: FJ652617.1). The strain was subsequently used for the *Demodex* survival inhibition experiment. In total, 30 *Demodex* mites were obtained from the eyelashes of 12 patients with DB other than those in the DB study group. Fifteen *Demodex* mites were cultured in the fermentation supernatant of the liquid medium with the *B. cepacia* culture, and another 15 *Demodex* mites were cultured in the liquid medium without the *B. cepacia* fermentation supernatant (Fig. 6e). The *Demodex* mites were observed to survive in the fermentation supernatant of the *B. cepacia* for a mean (± SD) of 42.20 ± 9.45 h versus 70.6 ± 22.95 h in the control group (Additional file 1). The difference in survival time was significantly different between the two groups (*P* < 0.001, Student’s t-test) (Fig. 6f).

## Discussion

The stability of the ocular surface microenvironment is essential for maintaining ocular surface health, which in turn is closely related to a stable microbial community [20]. Bacteria are the most abundant group of ocular surface microbiota, and any imbalance in the bacterial communities plays a critical role in ocular surface diseases [21]. When the homeostasis is disrupted by external or internal causes, the eye is most likely to develop infection and inflammation. DB is a common ocular surface disease, and current opinion is divided on whether bacteria are involved in its pathological process [22, 23]. *Demodex* infestation of the eyelid margin may contribute to bacterial infection [16], and it has been shown that the bacterial content of eyelashes from patients with blepharitis is higher than that of eyelashes from healthy people [15]. Therefore, it is relatively accepted that bacterial involvement in the pathological process of DB mainly uses *Demodex* mites as a carrier [17].

The traditional bacterial culture techniques used to evaluate DB bacterial communities have many limitations [24], such as the different culture conditions required for different bacteria, difficulties in culturing certain bacteria, the long culture cycle and, in some cases, special samples, such as eyelashes. With the continuous development of gene sequencing technology, 16S rDNA-based gene sequencing avoids the traditional culture methods and has gradually become an option for bacterial detection due to its high sensitivity, excellent comprehensiveness and the need for only a small sample size. Gene sequencing technology has been applied in the visual science field for many years [25, 26]. In the present study, we preferred eyelash tissue as samples for sequencing because eyelash follicles have a higher rate of *Demodex* infestation than other ocular tissues. The detection of bacterial communities in eyelash tissue is helpful towards determining the correlation between bacterial community imbalance and *Demodex* infestation. We also disinfected the eyelid skin, eyelid margins and eyelash shafts before obtaining samples to minimize the influence of long-term exposure to the external environment. The results from 16S rDNA gene sequencing did not reveal any difference in the species diversity, species richness and evenness of bacteria between the patient group and the healthy volunteer group. The separation trend of bacteria was not obvious, and the composition similarity was high.

A previous study using 16S rDNA sequencing to detect the eyelash bacterial community indicated that the genera Proteobacteria, Actinobacteria, Firmicutes, Bacteroidetes and Cyanobacteria dominated the bacterial communities in both patients with blepharitis and healthy individuals [18], which is highly consistent with our findings. We also observed that the abundance of *Actinobacteria* in the eyelash tissue of patients with DB was significantly upregulated. The lack of previous observations on the upregulation of *Actinobacteria* may be attributed to the insufficient depth of prior detection techniques or small sample sizes. The upregulation of *Actinobacteria* may provide a new basis for the use of tetracycline and other antibacterial drugs in the treatment of DB [17].

We did not find any significant difference in the abundance of *Propionibacterium* and *Staphylococcus* between the DB group and the healthy control group, as also described by Dougherpy et al. and Groden et al. [27, 28]. In another study, *Pseudomonas* was reported to be cultivated from samples of patients with blepharitis [29], but there was no difference from the samples collected from healthy individuals [18], which is confirmed by our results. Additionally, we found that *Burkholderia-Caballeronia-Paraburkholderia* was the only biomarker among the predominant bacterial species of both groups, being significantly higher in the control group than in the DB group. ROC survival curve analysis showed that the *Burkholderia-Caballeronia-Paraburkholderia* genus may have a certain diagnostic significance. The corresponding β-Proteobacteria order, *Burkholderia* family and *Burkholderia-Caballera-Paraburkholderia-Unclassified* species also showed biological significance, with significant differences between groups, as well as a certain diagnostic value for the disease. *Caballeronia* and *Paraburkholderia* are new bacterial genera isolated from the *Burkholderia* genus. *Burkholderia*, which is widely seen in water, soil, rhizosphere, insects and fungi [30, 31], was originally classified into genus *Pseudomonas* and was removed from genus * Pseudomonas* in 1992 [32]. This may explain why previous reports disclosed differences in *Pseudomonas* but not in *Burkholderia* [18, 29]. *Burkholderia* has also been found to secrete a variety of proteolytic enzymes and lipolytic enzymes, and some *Burkholderia* strains can produce antibiotics [33]. A new bacterial strain named *Burkholderia rinojensis SP. nov.* that can kill mites was isolated in 2013 [34]. To some extent, these functions explain our finding that the *Burkholderia* genus may be helpful to reduce the incidence of DB [3, 35]. We verified the effect of *Burkholderia* on *Demodex* activity and/or survival in patients with DB and obtained similar results as those reported previously. Our study is the first to show that the fermentation supernatant of *Burkholderia* is able to decrease the survival time of *Demodex* mites. To date, at least two *Burkholderia* strains have been reported to inhibit nematodes or mites [34, 36]. Although we verified the effect of *Burkholderia*, the positive rate of isolating it from healthy human eyelashes was low, which we believe may be related to the treatment method and culture method of the eyelash samples.

The causal relationship between infestation by *Demodex* mites and blepharitis remains unclear. Some investigators believe that inflammation caused by lipid accumulation and composition changes could promote *Demodex* infestation, while others consider that *Demodex* infestation may lead to inflammation. The authors of a recent study reported that changes in composition of the sebaceous gland could affect the bacterial community of the skin hair follicle microenvironment, resulting in abnormal local inflammation [37]. This finding in the skin may support the former theory. However, based on our findings and the biological role played by *Burkholderia*, it appears more plausible that imbalances of the bacterial community led to lipid accumulation and *Demodex* infestation.

 In summary, *Burkholderia* appeared to play a positive protective role in reducing *Demodex* infestation, lipid accumulation and inflammation. Acaricidal drugs prepared from the inactivated *Burkholder *strain A396 have been marketed in for agricultural applications and have achieved good effects [38]. This study also provides a reference for the prevention and treatment of human mite infectious diseases, including DB. Further investigations may focus on isolating *Burkholderia* from eyelashes and extracting the active ingredients from *Burkholderia* for the treatment of *Demodex* infestation.

## ﻿Conclusions

In conclusion, in comparison to the eyelashes of healthy individuals, the number of bacterial communities in eyelash follicles of DB patients decreased, the abundance of *Actinobacteria* increased significantly and the abundance of *Burkholderia* decreased significantly. These results further confirm an imbalance in the bacterial communities in the eyelashes of DB patients. The potential protective effect and diagnostic value of *Burkholderia* were initially revealed, as well as the possibility of anti-mite drugs based on *Burkholderia* to treat DB.

### Supplementary Information

**Additional file 1. ** Supplementary materials for identification of Burkholderia and survival time of each Demodex.

## Data Availability

All the raw and processed scRNA-seq files in this research have been deposited in the GSA database under accession code: CRA014100.

## Abbreviations

AUC: Area under the curve
DB: *Demodex* blepharitis
LEfSe: Linear discriminant analysis Effect Size
ROC: Receiver operating characteristic curve
