# Supplementary material for: Characteristics of bacterial community in eyelashes of patients with Demodex blepharitis
Source: Parasit Vectors. 2024 Feb 14;17:64. doi: 10.1186/s13071-024-06122-x (PMC10868039; doi:10.1186/s13071-024-06122-x)
Supplement: Supplementary file 1 — Additional file 1. Supplementary materials for identification of Burkholderia and survival time of each Demodex. [file 13071_2024_6122_MOESM1_ESM.docx]

**Supplemental material 1: Mass spectrometry results for *Burkholderia***

**
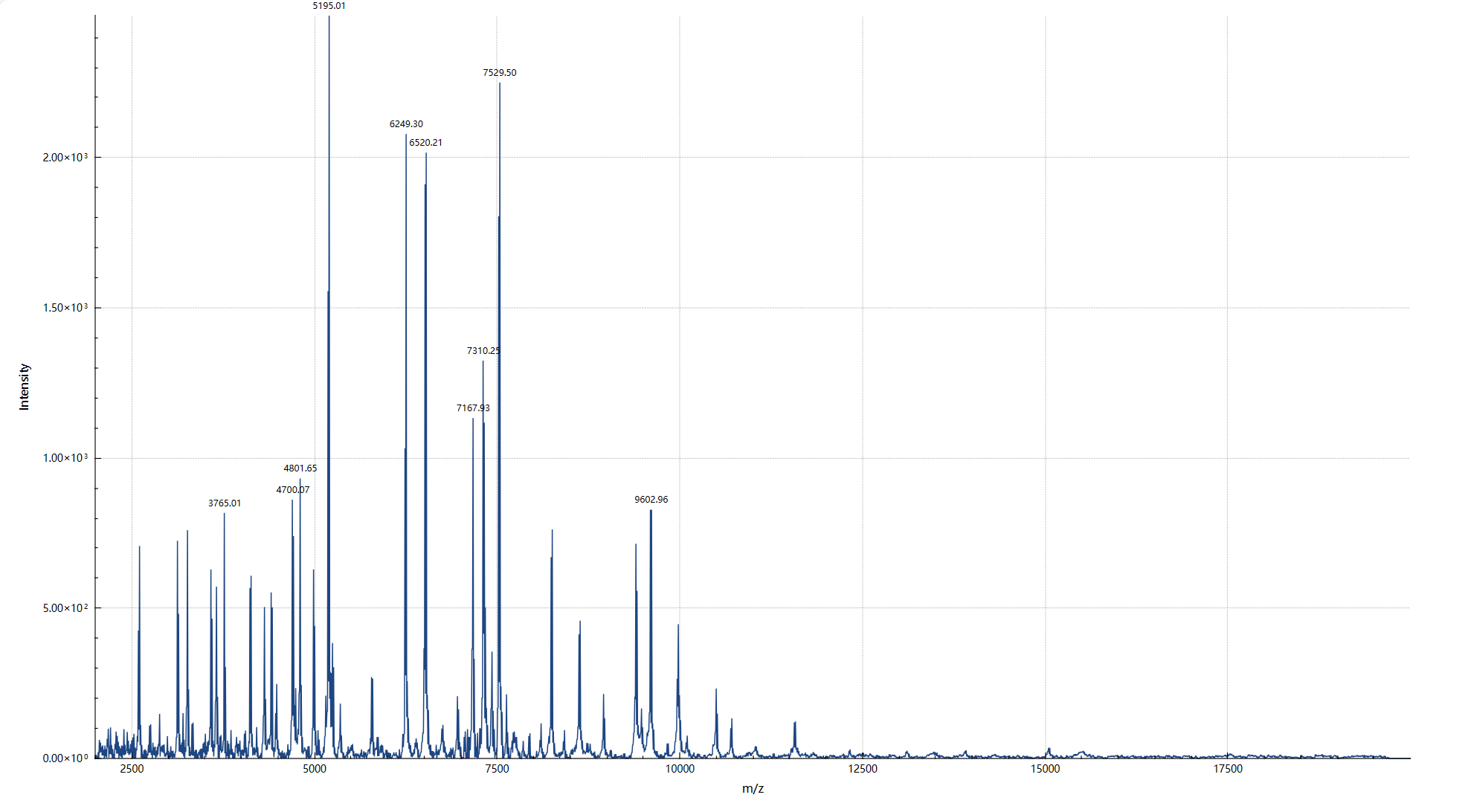
**

**Supplemental material 2: Nucleotide Sequence of** **the isolated-*Burkholderia:***

AAGTCGAACGGCAGCACGGGTGCTTGCACCTGGTGGCGAGTGGCGAACGGGTGAGTAATACATCGGAACATGTCCTGTAGTGGGGGATAGCCCGGCGAAAGCCGGATTAATACCGCATACGATCTACGGATGAAAGCGGGGGACCTTCGGGCCTCGCGCTATAGGGTTGGCCGATGGCTGATTAGCTAGTTGGTGGGGTAAAGGCCTACCAAGGCGACGATCAGTAGCTGGTCTGAGAGGACGACCAGCCACACTGGGACTGAGACACGGCCCAGACTCCTACGGGAGGCAGCAGTGGGGAATTTTGGACAATGGGCGAAAGCCTGATCCAGCAATGCCGCGTGTGTGAAGAAGGCCTTCGGGTTGTAAAGCACTTTTGTCCGGAAAGAAATCCTTGGTTCTAATACAGCCGGGGGATGACGGTACCGGAAGAATAAGCACCGGCTAACTACGTGCCAGCAGCCGCGGTAATACGTAGGGTGCGAGCGTTAATCGGAATTACTGGGCGTAAAGCGTGCGCAGGCGGTTTGCTAAGACCGATGTGAAATCCCCGGGCTCAACCTGGGAACTGCATTGGTGACTGGCAGGCTAGAGTATGGCAGAGGGGGGTAGAATTCCACGTGTAGCAGTGAAATGCGTAGAGATGTGGAGGAATACCGATGGCGAAGGCAGCCCCCTGGGCCAATACTGACGCTCATGCACGAAAGCGTGGGGAGCAAACAGGATTAGATACCCTGGTAGTCCACGCCCTAAACGATGTCAACTAGTTGTTGGGGATTCATTTCCTTAGTAACGTAGCTAACGCGTGAAGTTGACCGCCTGGGGAGTACGGTCGCAAGATTAAAACTCAAAGGAATTGACGGGGACCCGCACAAGCGGTGGATGATGTGGATTAATTCGATGCAACGCGAAAAACCTTACCTACCCTTGACATGGTCGGAATCCTGCTGAGAGGTGGGAGTGCTCGAAAGAGAACCGATACACAGGTGCTGCATGGCTGTCGTCAGCTCGTGTCGTGAGATGTTGGGTTAAGTCCCGCAACGAGCGCAACCCTTGTCCTTAGTTGCTACGCAAGAGCACTCTAAGGAGACTGCCGGTGACAAACCGGAGGAAGGTGGGGATGACGTCAAGTCCTCATGGCCCTTATGGGTAGGGCTTCACACGTCATACAATGGTCGGAACAGAGGGTTGCCAACCCGCGAGGGGGAGCTAATCCCAGAAAACCGATCGTAGTCCGGATTGCACTCTGCAACTCGAGTGCATGAAGCTGGAATCGCTAGTAATCGCGGATCAGCATGCCGCGGTGAATACGTTCCCGGGTCTTGTACACACCGCCCGTCACACCATGGGAGTGGGTTTTACCAGAAGTGGCTAGTCTAACCGCAAGGAGGA

**Supplemental material 3. The survival time of two groups of demodex.**

| Control Group | | Fermentation Supernatant Group | |
| --- | --- | --- | --- |
| Number | Survival Time (Hours) | Number | Survival Time (Hours) |
| 1 | 48 | 1 | 48 |
| 2 | 81 | 2 | 24 |
| 3 | 54 | 3 | 30 |
| 4 | 54 | 4 | 54 |
| 5 | 63 | 5 | 51 |
| 6 | 57 | 6 | 33 |
| 7 | 111 | 7 | 36 |
| 8 | 54 | 8 | 45 |
| 9 | 60 | 9 | 39 |
| 10 | 51 | 10 | 51 |
| 11 | 60 | 11 | 36 |
| 12 | 99 | 12 | 39 |
| 13 | 90 | 13 | 57 |
| 14 | 60 | 14 | 42 |
| 15 | 117 | 15 | 48 |
